# Hospital Questions Which Press

**Published:** 1905-03-25

**Authors:** 


### HOSPITAL ADMINISTRATION.

### CONSTRUCTION AND ECONOMICS.

### HOSPITAL QUESTIONS WHICH PRESS.

#### WANT OF CO-OPERATION.

(*Concluded from page* 394)

Remedies.

Few, if any, serious attempts have as yet been made to establish hospital co-operation on a sound basis; possibly the explanation of this lies in the principle that everybody's business is nobody's business. But it is with the managers of the voluntary hospitals, and the medical schools attached to them, that the responsibility lies, and on them the onus of reform undoubtedly falls. And, to commence with, we should like to advocate four measures of reform. They are—

1. Affiliation of special with general hospitals.
2. The establishment of adequate facilities for telephonic communication between the various hospitals.
3. The abolition of appeals to the general public on the part of individual hospitals.
4. The establishment of a better system of preliminary and advanced medical instruction.

Affiliation of Special with General Hospitals.

Some years ago it was suggested that if certain of the special hospitals were to become amalgamated with general hospitals considerable economy would be thereby effected, while other advantages would be gained by both contracting parties. So far the difficulties in the way of carrying out this scheme have proved too much for the hospital managers. The case of Charing Cross Hospital and the Westminster Ophthalmic Hospital illustrates this. The two hospitals together occupy the whole of a triangular area which is not intersected by a street. The Westminster Ophthalmic Hospital occupies one angle of the triangle, Charing Cross Hospital occupies the remainder. Strenuous endeavours were made not long ago to amalgamate the two, but without success, and the attempt was definitely abandoned by the managers of the two hospitals as hopeless. We understand that the failure to effect a satisfactory settlement was chiefly due to the great and obvious difficulty of dealing successfully with the question of the vested interests of the two parties. Now it is clear that Charing Cross Hospital must have an ophthalmic department in order to be fully efficient. It is also obvious that the needs of the public do not require two separate institutions for the treatment of diseases of the eye which are adjacent to one another. Here there is a waste of public money actually going on. It must be so. To work two institutions with two separate staffs where one could do the work is obviously to abuse public charity. This is a matter of urgency, and a committee of inquiry, instituted by the managers of King Edward's Hospital Fund to thoroughly investigate the obstacles which have in this particular case rendered affiliation impossible, would be welcomed by the public. It is more than likely that similar difficulties will be met with in other attempts at amalgamation, and if it could perchance be shown that, after all, in this case the obstacles might be circumvented a most important precedent would have been established which might ultimately be of the greatest value in assisting the union of many other hospitals which are at present working independently of each other. Let this be a test case on the result of which subsequent efforts of a similar nature may be bound.

Telephonic Communication between Hospitals.

The second remedy advocated is a matter of the greatest importance, and is one which is not surrounded by any great difficulties. By the free use of such facilities much suffering, expense, and unnecessary delay would be saved. The means whereby such a scheme may be rendered effective are already to hand. The Hospitals Association, 28 and 29 Southampton Street, Strand, would be quite willing to serve as a central office. The plan suggested is this: Every day each hospital would telephone to the central office how many beds it had vacant, stating whether in male or female, or medical or surgical wards. As the beds became occupied, the fact would be notified by telephone to the central office; or if this were found unworkable, the central office, on being questioned, would telephone and find out where there was a vacant bed. In this manner, if a patient urgently requiring admission could not be given a bed at the first hospital at which he was seen, it could be ascertained in a few minutes where he could be admitted, and he might thereby be saved much discomfort, disappointment, and even danger. This plan appears to be so simple and easy of adoption, and at the same time it might be rendered so effective and useful, that we trust it will be well considered in every point by the managers of the general hospitals throughout London.

Abolition of General Appeals by Individual Hospitals.

It would be an important step in the right direction if the custom of individual hospitals appealing to the general public of London for support were to be abandoned. Appeals to the general public should be confined to appeals on behalf of the whole of the hospitals requiring help. Each separate hospital may well confine its energies to its own immediate vicinity in attempting to obtain support. In this way many evils would disappear. Hospitals would cease to be in competition either for patients or for funds, and these two unhealthy forms of rivalry would cease to exist.

Co-operation of Medical Schools.

The last change which is advocated has reference to the medical schools, and the remedy rests with the medical school authorities. Instead of the present arrangements for medical instruction, such a system as exists in Paris could be instituted. In Paris students can go and attend any hospital they like, but they get their medical instruction at certain centres where they have the very best teachers. In London they must get their instruction at their own hospitals, where they cannot always get the best teaching. They may have plenty of patients there, but the physicians and surgeons are often too busy to give up sufficient time to teaching. In Paris certain men when they get to be in a leading position give up a very large portion of their time to teaching, whereas in London the teachers are men who are engaged in active practice and are not able to give all the time that is necessary to teaching. It would be a great service if the students of our hospitals were enabled to attend the clinics at other hospitals, whereby the abilities of individual teachers would be rewarded, and the opportunities for receiving instruction would be considerably increased.

### Conclusion.

It would be highly irrational to expect that a complete cure of the evils which have been mentioned could by any means be effected immediately, and without delay or difficulty. The magnitude of the hospital system of London is in itself an obstacle, and the reforms which are necessary are of a fundamental nature, and must take a long time even to initiate. Old traditions have to be overcome, to accomplish which there is nothing which requires greater determination. But the patience, the hard labour, and the long time expended in achieving a result cannot be rightly put forward as reasons for letting an evil system continue and not attempting any reform. The suggestions we have made are not presumed either to be easy of application or to completely abolish the evils which now exist. It is merely claimed for them that they are not unreasonable or impossible, and that they are advances in the right direction. If everyone will give his support much may be effected. If those who desire to help the voluntary hospitals, and who have no knowledge of or interest in any particular hospital, will in the future give their subscriptions and donations to one of the central hospital funds, such as the King's Hospital Fund or the Hospital Sunday Fund, they will be doing a considerable service, not only to each voluntary hospital, but to the entire system of medical charity in London. By adopting such a course they could feel assured that their contributions would ultimately find their way to those institutions which were most deserving and most in need. And it is more than probable that the managers of these central funds will find themselves in each successive year in a better position to judge impartially the comparative worth of the claims put forward by the different institutions, and that, in consequence, the appeals made by each hospital will have to be based, in order to be successful, on the quality of its work and economy of management, rather than upon the number of patients it attends to in a year or the number of cough lozenges these patients devour. The duty which falls upon all classes of the public is quite clear: if they will follow the course here indicated the need for the existing arrangements, whereby the voluntary hospitals are apparently in open competition for public charity, will cease, and the public will have a guarantee that its gifts will find their way to the most deserving quarters where they will be in no measure wasted or misapplied. In addition to these advantages the managements of the central hospital funds will be strengthened and placed in a better position to benefit the individual hospitals by advice and guidance, based on inquiry and experience, which the past few years have shown to be so beneficial to these institutions, their patients and managers alike.

